# Modelling the benefits of long-acting or transmission-blocking drugs for reducing *Plasmodium falciparum* transmission by case management or by mass treatment

**DOI:** 10.1186/s12936-017-1988-4

**Published:** 2017-08-16

**Authors:** Michael T. Bretscher, Jamie T. Griffin, Azra C. Ghani, Lucy C. Okell

**Affiliations:** 1 Department of Infectious Disease Epidemiology, MRC Centre for Outbreak Analysis & Modelling, Imperial College, London, UK; 2 School of Mathematical Sciences, Queen Mary University of London, London, UK; 3 F. Hoffmann-La Roche Ltd, Basel, Switzerland

**Keywords:** Mathematical modelling, Transmission, Treatment, Anti-malarial, Mass drug administration, Primaquine, Artemisinin combination therapies, Prophylaxis

## Abstract

**Background:**

Anti-malarial drugs are an important tool for malaria control and elimination. Alongside their direct benefit in the treatment of disease, drug use has a community-level effect, clearing the reservoir of infection and reducing onward transmission of the parasite. Different compounds potentially have different impacts on transmission—with some providing periods of prolonged chemoprophylaxis whilst others have greater transmission-blocking potential. The aim was to quantify the relative benefit of such properties for transmission reduction to inform target product profiles in the drug development process and choice of first-line anti-malarial treatment in different endemic settings.

**Methods:**

A mathematical model of *Plasmodium falciparum* epidemiology was used to estimate the transmission reduction that can be achieved by using drugs of varying chemoprophylactic (protection for 3, 30 or 60 days) or transmission-blocking activity (blocking 79, 92 or 100% of total onward transmission). Simulations were conducted at low, medium or high transmission intensity (slide-prevalence in 2–10 year olds being 1, 10 or 40%, respectively), with drugs administered either via case management or mass drug administration (MDA).

**Results:**

Transmission reductions depend strongly on deployment strategy, treatment coverage and endemicity level. Transmission-blocking was most effective at low endemicity, whereas chemoprophylaxis was most useful at high endemicity levels. Increasing the duration of protection as much as possible was beneficial. Increasing transmission-blocking activity from the level of ACT to a 100% transmission-blocking drug (close to the effect estimated for ACT combined with primaquine) produced moderate impact but was not as effective as increasing the duration of protection in medium-to-high transmission settings (slide prevalence 10–40%). Combining both good transmission-blocking activity (e.g. as achieved by ACT or ACT + primaquine) and a long duration of protection (30 days or more, such as provided by piperaquine or mefloquine) within a drug regimen can substantially increase impact compared with drug regimens with only one of these properties in medium to high transmission areas (slide-prevalence in 2–10 year olds ~10 to 40%). These results applied whether the anti-malarials were used for case management or for MDA.

**Discussion:**

These results emphasise the importance of increasing access to treatment for routine case management, and the potential value of choosing first-line anti-malarial treatment policies according to local malaria epidemiology to maximise impact on transmission. There is no indication that the optimal drug choice should differ between delivery via case management or MDA.

**Electronic supplementary material:**

The online version of this article (doi:10.1186/s12936-017-1988-4) contains supplementary material, which is available to authorized users.

## Background

Anti-malarial drugs are increasingly recognised as an important tool for reducing *Plasmodium falciparum* malaria transmission, as well as their vital function in treating clinical cases and clearing the blood-stage infection which causes symptoms. Drug discovery and development, however, are costly and time consuming, and identifying the most promising candidate compounds and product profiles early in the development process is, therefore, crucial [1, 2].

Anti-malarials are typically used in one of three ways: (A) for clinical management of symptomatic cases delivered by the public health system, private providers or community health workers (referred to here as case management, CM); (B) to protect against infection in at-risk groups including in infants and pregnant women (intermittent preventive treatment, IPTi and IPTp) young children residing in areas with seasonal malaria (seasonal malaria chemoprevention, SMC) and in travellers from non-endemic areas (chemoprophylaxis); and (C) to clear the parasite reservoir in endemic populations as a means to interrupt transmission either as mass drug administration (MDA) or via mass or focal screen and treat (MSAT, FSAT) [3, 4].

The primary effect of an anti-malarial drug within the human host is to kill the parasite’s blood stage or inhibit its replication in order to reduce the in-host parasite population size to zero. In addition, drug compounds which have a long elimination half-life in the body may provide protection against re-infection for the period of time that drug concentrations remain sufficiently high to prevent parasite replication. Finally, some anti-malarial compounds possess gametocytocidal activity, which reduces onward transmission to mosquitoes by additionally killing the sexual stages of the parasite. These can otherwise remain in the host for several weeks after clearance of the blood stage infection and continue to infect mosquitoes [5].

It can be assumed that every newly developed drug would be required to demonstrate efficacy in clearing infections at a level comparable to current artemisinin-based combination therapy (approximately 95% or higher in areas without drug resistance [6]). The second action of the anti-malarial, chemoprevention, may provide additional benefits to the treated individual by protecting them from future clinical attacks. Both these modes of action will also reduce onward transmission and, therefore, have a population impact. Transmission-blocking activity, by contrast, has no direct benefit to the treated patient but acts solely at the population level by reducing transmission.

To make decisions on which compounds to prioritise during the drug development process, an understanding of the relative importance of transmission-blocking activity compared to chemoprevention is needed. Furthermore, it is unclear whether different drug properties may be required for anti-malarials being used for case management from those used in interventions aiming to reduce transmission. Here an existing model of the transmission of *Plasmodium falciparum* malaria was extended to explore the potential impact of these different drug properties to reduce and interrupt transmission. Previous modelling analyses have estimated the impact of specific anti-malarials on within-host dynamics, individual protection and transmission intensity [7–16]. Here, previous work is extended by (a) looking at a wide range of potential drug action, including different combinations of transmission-blocking activity and prophylactic effects, to cover products in development rather than only specific existing anti-malarials and (b) contrasting what drug properties are most beneficial under two usage scenarios—clinical case management (CM) and mass drug administration (MDA), both in the context of an individual-based malaria transmission model that has been fitted to a wide variety of data from malaria-endemic areas [11, 17].

## Methods

**Fig. 1:**
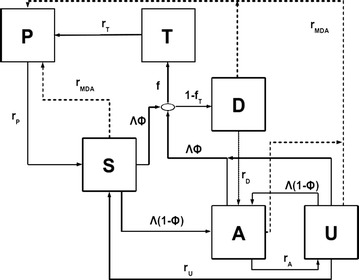
Model of malaria epidemiology. A modified version of the *P. falciparum* transmission model detailed in [11, 17] was used to simulate the impact of treatment. Human hosts may be in state S (susceptible), D (diseased not treated), T (diseased, destined to be treated after 5 days), P (under chemoprophylaxis), A (carrying asymptomatic infection), or U (carrying undetectable infection). Recovery rates are denoted by r with the state of origin as subscript; an exception is recovery due to MDA which is indicated as r_MDA_. Further parameters are the force of infection Λ, the fraction of blood-stage infections that result in clinical symptoms Φ, and the fraction of clinical cases treated f_T_. The model additionally includes age structure, immunity and heterogeneous exposure to mosquito bites (not shown)

A previously described age-structured mathematical model of *P. falciparum* epidemiology was used to simulate the impact of drug administration on malaria transmission [11, 17]. The original model was parameterized by fitting to data from a wide variety of endemic settings. However, age-stratified data on human infectivity to mosquitoes were only available from high transmission locations. To allow exploration of the impacts of drug administration at low transmission, the model was modified as detailed below to better capture infectivity in populations with low immunity (Fig. 1). The main changes were made with respect to the transmission output of treated infections to match a more detailed model of the within-host dynamics of malaria over time [7]. In the original model, susceptible individuals (S) become infected at a rate given by the force of infection from mosquitoes. Following a delay to account for the liver stage of infection, individuals then proceeded to either a clinical disease state (D) or asymptomatic state (A) depending on their level of immunity (which develops dynamically as detailed in [17]) with those that are asymptomatic moving to a sub-microscopic stage U after a period of time. The onward infectivity to mosquitoes is highest in the disease state D, moderate in the asymptomatic state A and lowest in the sub-microscopic state U, to capture patterns of onward infectivity from feeding studies. In the revised model, newly symptomatic cases that will seek care enter the treated state T with a mean duration of 5 days to reflect an approximate estimated period between development of symptoms and receiving treatment, considering times reported in under-5 year old children (~0 to 2 days in surveys from multiple endemic sub-Saharan countries available at [18]) and the likely longer waiting time in adults. During this time period they retain the same level of infectivity as symptomatic cases that do not seek care. Following the administration of drugs (via either treatment of disease or administration to those with asymptomatic infection), with the probability p_eff_ that the drug is efficacious (assumed to be 0.97 here in all simulations), individuals enter one of three “protected” states P: P_T_, P_DAU_, and P_S_ (for simplicity summarized as P in Fig. 1) where they are protected from re-infection. The subscripts T, DAU, and S indicate the states from which freshly treated individuals originate to reflect varying levels of pre-treatment human-to-mosquito infectiousness. Individuals in the protected P states remain infectious to mosquitoes with a reduction in total infectivity over the whole infectious period dependent on the transmission-blocking effect of the drug they receive (this was achieved by scaling infectivity according to the duration of prophylaxis). The parameters c_PT_ and c_PDAU_ denote the probability of a mosquito becoming infected upon biting a person in the respective states P_T_ and P_DAU_. The full equations for the model and a list of the key parameters are given in Table 1 and Additional file 1.

**Table 1:** Key model parameters for describing the effect of treatment on transmission

Description	Symbol	Value(s)	References
Untreated infections			
Mean duration of state (days)			[17]
D (clinical disease)	d_D_	5	
A (asymptomatic)	d_A_	195	
U (submicroscopic)	d_U_	84	
Probability of human-to-mosquito transmission per mosquito bite from individuals in state			[17]
D (clinical disease)	c_D_	0.0868596	
A (asymptomatic)	c_A_	c_D_ > c_A_ > c_U_ varies by immunity	
U (submicroscopic)	c_U_	0.0023444	
Total infectivity over the infectious period in untreated, initially symptomatic infections	O_tot_	c_D_d_D_ + c_A_d_A_ + c_U_d_U_	–
Proportion of infections which develop symptoms	φ	0.00038–0.81 (immunity-dependent)	
Treated infections			
Drug efficacy: probability the drug eventually clears parasites	p_eff_	0.97	[6]
% reduction O_tot_ in total infectivity over the infectious period achieved by treatment with:			[17]
SP	O_red_	79.1%	
ACT		92.7%	
ACT + primaquine		100%	
Mean duration of state			[17]
P_DT_, P_AU_, or P_S_ (prophylaxis)	d_P_	3, 30, 60	
T	d_T_	5	
Probability of human-to-mosquito transmission per mosquito bite from individuals in state			This paper
T (will soon receive treatment)	c_T_	c_D_	
P_DT_ (prophylaxis)	c_PDT_	O_tot_ * (1 − O_red_/100)/d_P_	
P_AU_ (prophylaxis)	c_PAU_	c_A_ * c_PDT_/c_D_	
MDA coverage: proportion of individuals receiving MDA	f_MDA_	0.8	–
Case management coverage: proportion of cases treated	f_T_	0.4–1	–

A full parameter list is given in [17]

The total infectivity, O_tot_, of an untreated infection which is initially symptomatic is therefore given by

$${\text{O}}_{\text{tot}} = {\text{c}}_{\text{D}} {\text{d}}_{\text{D}} + {\text{c}}_{\text{A}} {\text{d}}_{\text{A}} + {\text{c}}_{\text{U}} {\text{d}}_{\text{U}}$$

where d denotes the duration in each state, with states shown as subscripts, and c_A_ (in asymptomatics) is identical to c_D_ in the case of non-immune hosts and decreases with increasing levels of immunity.

Proportional reductions in total infectiousness O_red_ attained by treating a non-immune patient for three different drug combinations with increasing gametocytocidal effect were simulated. There is considerable uncertainty over the exact impact of different drugs, mainly because it is difficult to get accurate measures of the total infectivity of untreated infections in endemic areas. This parameter was varied, choosing values approximately corresponding to particular drugs. Two of these drug effects were estimated from analysis in [7]: SP giving 79.1% reduction and ACT 92.7% reduction. This analysis was a within-host model which explicitly included gametocytes and drug effects upon gametocytes, and estimated reductions in infectivity using a relationship between gametocyte density and infectiousness to mosquitoes. SP is assumed to clear blood-stage infection without killing gametocytes. Increasing gametocytocidal activity using artemisinin derivatives, which kill immature gametocytes, further reduces the reproductive output of an infection. The previous modelling analysis, like others (including our previous work) [14, 19], had also estimated the impact of ACT + primaquine using gametocyte densities, at around 94% reduction in total onward infectiousness. However since this analysis was done, further data from human-to-mosquito transmission studies suggest a much larger effect of ACT + primaquine, close to 100% reduction in transmission in areas without artemisinin resistance [20–22]. Estimates of primaquine effect based on gametocytaemia are now believed to be too low because primaquine appears to sterilise gametocytes much more rapidly than it kills them, and may also disproportionately affect male gametocytes, producing a much larger reduction in onward transmission to mosquitoes than in gametocytaemia [23–25]. Based on this evidence, a drug was simulated which was 100% effective in blocking transmission immediately after treatment, to represent the effect of a perfect transmission blocker (now thought to approximately represent ACT + primaquine). The total infectivity of a freshly treated infection can be written in the two equivalent ways on either side of the following equation,

$${\text{c}}_{\text{PDT}} {\text{d}}_{\text{P}} + {\text{c}}_{\text{T}} {\text{d}}_{\text{T}} = {\text{O}}_{\text{tot}} \left( { 1- {\text{O}}_{\text{red}} } \right).$$

The human infectiousness in state T, c_T_ was set equal to c_D_, relative reductions in infectiousness O_red_ were entered for each drug and then the equation was solved for c_PDT_. It was assumed that the value of c_PDT_ for a semi-immune, freshly treated individual is identical to that of a non-immune, as both have recently experienced parasite densities high enough to trigger clinical symptoms. Hosts who clear infection after MDA and enter state P_DAU_, however, infect mosquitoes with a lower probability c_PDAU_. This parameter was determined such that the ratio c_PDAU_/c_A_ remained equal to c_PT_/c_D_, where c_A_ is the parameter that represents the infectivity of asymptomatically infected humans A to mosquitoes. Individuals in the P_DAU_ state originate from the D, A and U states, and the infectivity was adjusted relative to the A state because it has a much longer duration than either the D or U states (Table 1) and, therefore, the majority of individuals in the P_DAU_ state originate from A (70–79% in the settings chosen). We checked this assumption against simulations which weighted the infectivity of the P_DAU_ state according the proportion of individuals in the D versus A versus U state and obtained negligible change in results.

Drugs with three durations of post-treatment protection (3, 30 or 60 days) were compared. These values were selected to represent a range of prophylactic times with a high maximum value (e.g. the estimated durations of post-treatment protection for artemisinin monotherapy, lumefantrine and piperaquine are 0, 14, and 28 days, respectively [12]). To explore the impact of combined drug properties, the effect of all combinations of post-treatment protection and levels of transmission-blocking activity was also simulated.

Two modes of drug delivery were simulated: case management (CM), whereby a proportion f_T_ of symptomatic cases are treated, and mass drug administration (MDA), where treatment is given to a proportion of the population f_MDA_ without diagnostic testing. Two, 4, or 6 rounds (N_rounds_) of MDA treatment per year was simulated at evenly spaced intervals (e.g. 2 rounds per year at 6 month intervals), at 80% coverage and a drug with 97% efficacy at clearing parasites.

The relative reduction in clinical incidence among infants below 5 years of age was used to assess impact on transmission. This measure was chosen as it has an almost-linear relationship with changes in entomological measures of transmission intensity in the model except at high transmission levels [17] and at the same time is a direct indicator of impact on clinical burden. The reduction in clinical incidence in 0–5 year olds was calculated at the new endemic equilibrium reached after the drug intervention. The model was considered to have reached an equilibrium once there was <0.001 difference between successive annual average number of clinical episodes per 0–5 year old. In MDA simulations, the reduction in clinical incidence was calculated using the average clinical incidence at equilibrium over 5 years to smooth out the effects of MDA timing. Simulations were performed at three levels of malaria pre-treatment endemicity, characterized by the slide prevalence in 2–10 year olds (PR_2–10_). These levels were at a PR_2–10_ of 1, 10, and 40%.

To further assess the potential use of highly gametocytocidal drugs for elimination, in the simulations conducted at a pre-intervention PR_2–10_ 1% the time to elimination was recorded when a simulated 60–100% of clinical cases were treated with either ACT or ACT + primaquine. Elimination was defined as less than one infected person in 100,000.

## Results

### Case management

**Fig. 2:**
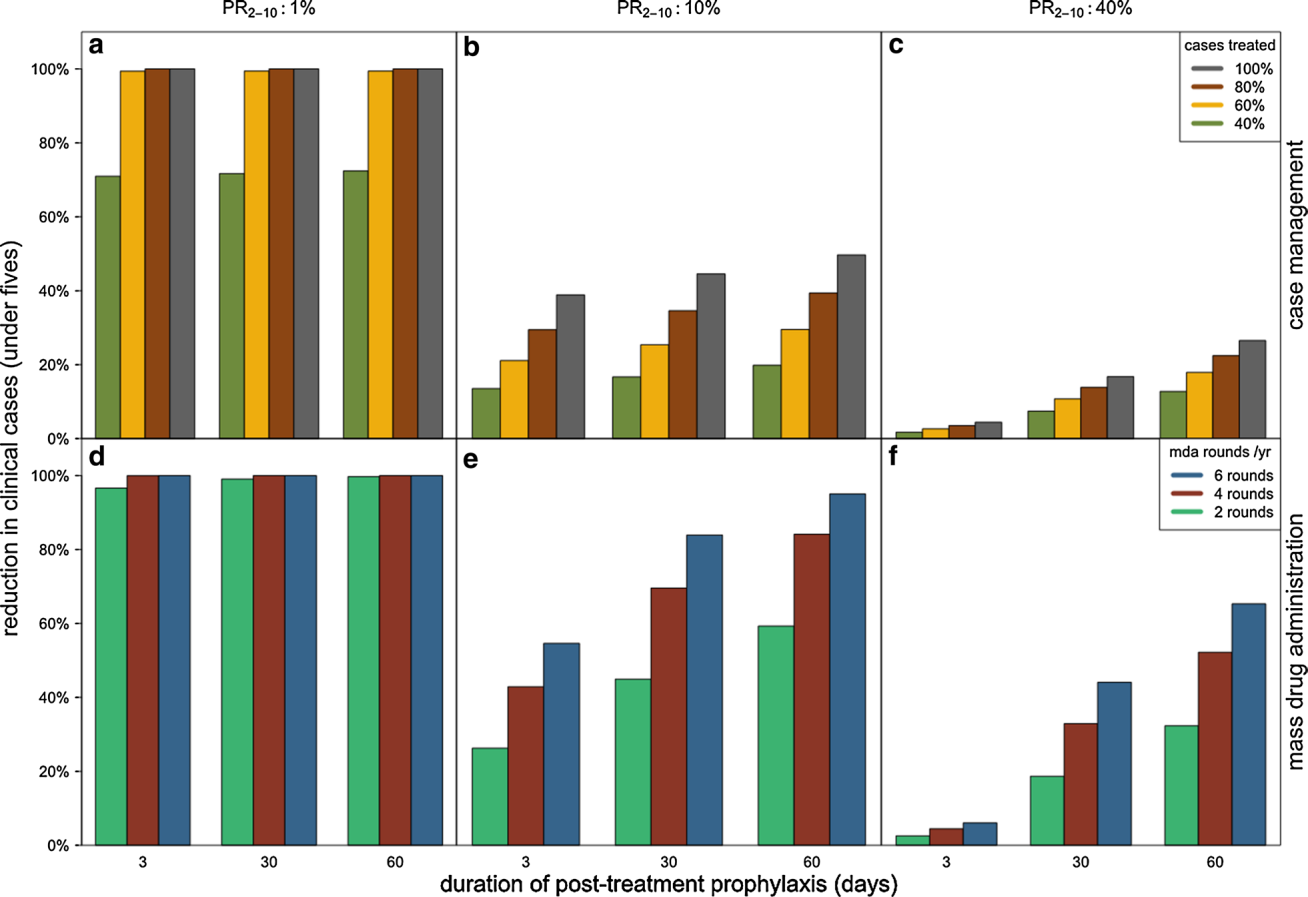
Effect of post-treatment protection on reductions in clinical malaria. Relative reduction in clinical incidence among children under 5 years due to case management (**a**–**c**) or MDA (**d**–**f**) with drugs of increasing duration of post-treatment protection (x-axis). Low, medium, and high endemicity levels (*columns*) are specified using prevalence among 2–10 year old children (PR_2–10_). The simulated drugs vary in the duration of prophylaxis but all have a low (SP-like) effect on onward infectivity. Case management (**a**–**c**): coverage levels (40, 60, 80, 100%) indicate the proportion of clinical malaria cases treated, and there is no MDA in these simulations. MDA (**d**–**f**): these transmission reductions are the result of long-term, sustained MDA to a randomly chosen 80% of the population 2, 4, or 6 times a year, assuming no case management

Overall, the least impact on onwards transmission is estimated by our model when a drug with the lowest transmission-blocking activity (i.e. SP-like) and a short duration of protection (here 3 days) is delivered via case management. However, even with such a drug, a substantial reduction in onward transmission is predicted even at moderate coverage levels in the simulated low transmission setting (PR_2–10_ of 1%) (Fig. 2). Here, the reduction in clinical cases in 0–5 year olds caused by case management is predicted to be greater than 90% at a coverage level of 60%, compared with no case management, when the model is run to equilibrium (Fig. 2a). At moderate transmission (PR_2–10_ of 10%), a more moderate impact of 21% reduction is predicted (Fig. 2b), whilst at high transmission (PR_2–10_ of 40%) the impact is limited to 3%. The model was also used to estimate how long it takes to achieve these reductions. 80% of the total ultimate reduction in clinical incidence is achieved within 7 months, 3.2 and 3.5 years in the high, medium and low transmission setting, respectively.

Additional transmission reduction is predicted when the duration of protection of the drug is increased (Fig. 2). The relationship between the duration of protection and transmission reduction is approximately linear and the highest impact is achieved at high coverage and high endemicity (Fig. 2). For example, in a moderate transmission setting, (PR_2–10_ of 10%) at 80% case management coverage the reduction in clinical cases in 0–5 year olds using a drug with a duration of protection of 60 days is estimated to be 1.4 times higher than when using a drug with a duration of protection of 3 days (assuming SP-like transmission-blocking activity in both scenarios), whereas in a high transmission setting (PR_2–10_ of 40%), the estimated impact is 5.6 times higher with the longer-acting drug. In the lowest transmission setting examined here (PR_2–10_ of 1%), increasing the duration of protection from 3 to 60 days had very little effect, with the estimated impact being only 1.02 times higher with the longer-acting drug.

**Fig. 3:**
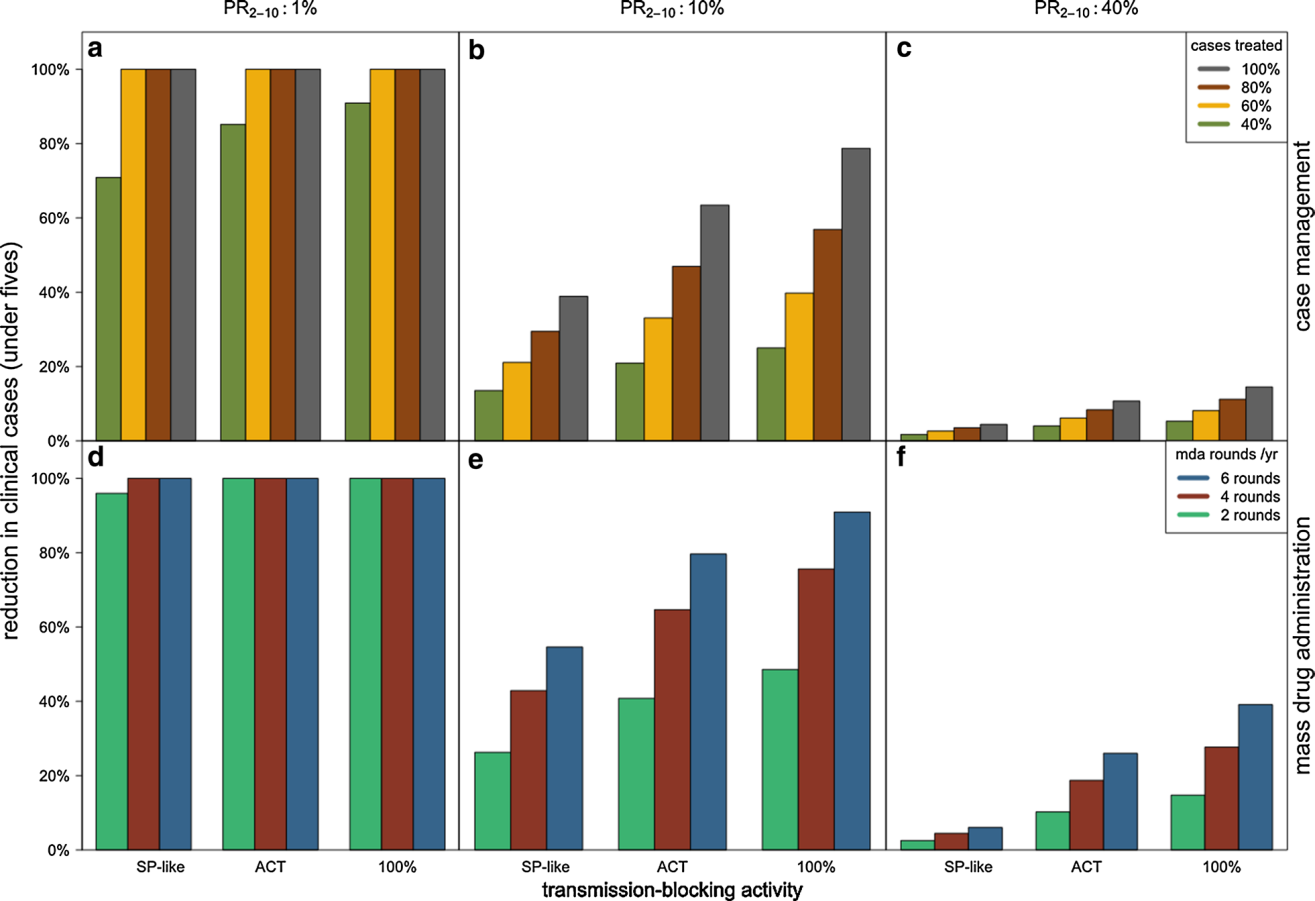
Effect of drugs with different levels of transmission-blocking activity on reductions in clinical malaria. Relative reduction in clinical incidence among children under 5 years due to case management (**a**–**c**) or MDA (**d**–**f**) with drugs of increasing transmission-blocking activity (x-axis). Low, medium, and high endemicity levels (*columns*) are indicated by slide prevalence among 2–10 year old children (PR_2–10_). The simulated drugs are short-lived (allowing re-infection after 3 days) but exhibit transmission-blocking activity comparable to sulfadoxine-pyrimethamine (SP), artemisinin combination therapies (ACT), or ACT + primaquine (100%). Case management (**a**–**c**): coverage levels (40, 60, 80, 100%) indicate the proportion of malarial fever cases treated, and impact shown is in the absence of MDA. MDA (**d**–**f**): the steady-state transmission reductions are the result of long-term, sustained MDA to a randomly chosen 80% of the population 2, 4, or 6 times a year, in the absence of any case management

Using a drug with a short duration of protection but with transmission-blocking activity comparable to current ACT (assumed to reduce total onward infectivity by 92.7%) is predicted to reduce transmission compared to non-gametocytocidal SP-like drugs (assumed to reduce total onward infectivity by 79.1%) (Fig. 3). In particular, in the simulated low transmission setting, the impact of increasing gametocytocidal activity from SP-like levels to current ACT-like levels in a drug used for case management is predicted to have a greater impact than increasing the duration of protection to 60 days. However, this relationship is reversed at high transmission, with a longer duration of protection providing greater benefit than increased transmission-blocking.

A perfect transmission-blocking drug which reduces onward transmission by 100% (as is thought to be close to the impact achieved by ACT + primaquine [20, 21, 26]) is predicted to increase impact over ACT-like transmission-blocking drugs (92.7% reduction in onward transmission) in all transmission settings, although the gain is not as large as increasing from SP-like transmission-blocking activity to ACT-like transmission blocking activity. For example, in the low transmission setting (*Pf*PR = 1%) when the duration of protection is 3 days and case management coverage is 40%, a 100% transmission-blocking drug is estimated to reduce clinical incidence by 91%, versus 85% for an ACT-like drug and 71% for an SP-like drug. In the medium-to-high simulated transmission settings, there was also a moderate gain from increasing from ACT-like to 100% transmission-blocking. For example, in the medium transmission setting (*Pf*PR = 10%) at 80% coverage of a drug with a short 3-day duration of protection, predicted impact increased from a 29% reduction with an SP-like drug, to 46% with an ACT-like drug and 56% with a perfect transmission blocker. The gain was similar in relative terms in the high transmission site under the same conditions, with 3.5% reduction with an SP-like drug, 8.4% reduction with an ACT-like drug and 11.1% with a perfect transmission blocker.

**Fig. 4:**
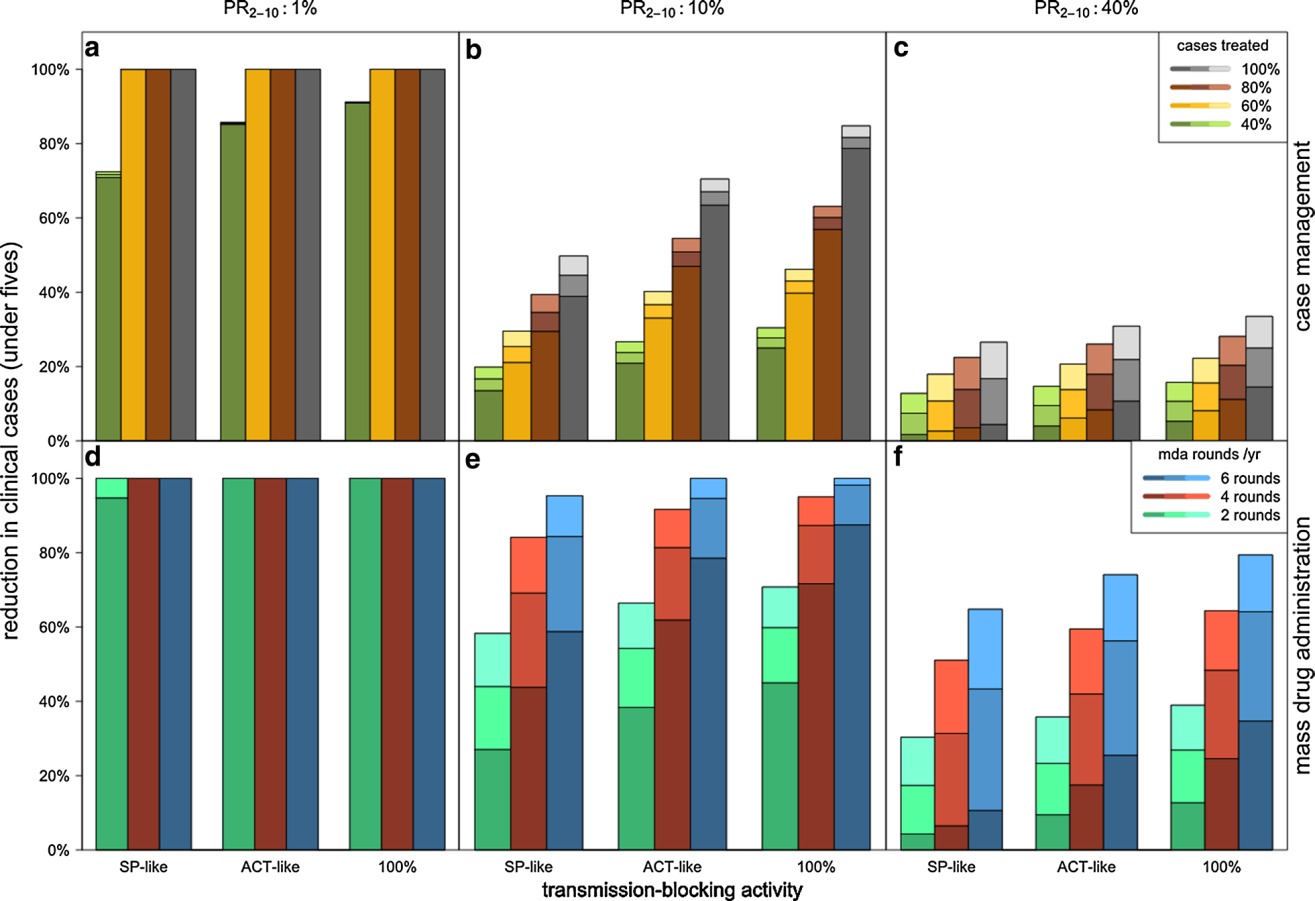
Combined effects of transmission-blocking and post-treatment protection on reductions in clinical malaria. Relative reduction in clinical incidence among children under 5 years due to case management (**a**–**c**) or MDA (**d**–**f**) with simulated drugs showing each combination of length of post-treatment protection (*stacked bars*, with the *lowest bars* and *darkest colours* indicating 3 days of protection, the *middle bar* showing 30 days of protection and the *highest bars* and *lightest colours* showing 60 days of protection) with different levels of transmission-blocking activity (x axis: activity comparable with sulfadoxine–pyrimethamine (SP), artemisinin combination therapies (ACT), or a 100% transmission blocker (comparable with ACT + primaquine). Low, medium, and high endemicity levels pre-intervention (*columns*) are indicated by slide prevalence among 2–10 year old children (PR_2–10_). Case management (**a**–**c**): coverage levels (40, 60, 80, 100%) indicate the proportion of malarial fever cases treated, and impact shown is in the absence of MDA. MDA (**d**–**f**): the transmission reductions are the result of long-term, sustained MDA to a randomly chosen 80% of the population 2, 4, or 6 times a year, in the absence of any case management

Combining drug properties so that a drug provides both a longer duration of protection and increased gametocytocidal activity is predicted to give greater impact compared with a drug which only has one of those properties in the medium–high transmission settings (PfPR = 10 and 40%) (Fig. 4). For all case-management scenarios examined in the moderate-high transmission settings, the effect of combining drug properties was very close to being exactly multiplicative of the effect of the individual drug properties (i.e. the clinical incidence rate ratio comparing a long-acting, highly transmission-blocking drug to a drug which provides 3 days protection and SP-like transmission-blocking activity is the same as multiplying together the rate ratios describing the reductions achieved by a drug which is only long-acting and a drug which is only transmission-blocking). For example, when PfPR = 10% and there is 60% coverage of case management, increasing the duration of protection alone from 3 to 30 days (keeping transmission-blocking activity at SP-like levels) produces an estimated 5% reduction in clinical cases. Increasing transmission-blocking activity alone from levels comparable with SP to levels comparable with ACT (keeping the duration of protection at 3 days) reduces clinical cases by an estimated 15%. Increasing both the duration of protection from 3 to 30 days and the transmission-blocking activity from SP-like to ACT-like levels is estimated to reduce clinical cases by 20%. In the lowest transmission scenario, there was negligible benefit of increasing the duration of protection in the presence of any level of transmission-blocking activity (Fig. 4a).

One postulated potential impact of additional transmission-blocking activity is that it could shorten the time required to eliminate malaria in low transmission settings. ACT + primaquine delivered via case management is predicted to shorten the time to elimination in low transmission settings compared with ACT alone (by approximately 15–30% for coverages of >60% at a starting PR_2–10_ of 1%, regardless of the duration of protection provided by the drug since it has negligible effect in low transmission settings). However, in all scenarios the coverage of the intervention has a greater estimated impact on elimination timescales than only increasing transmission-blocking effect. For example, increasing case management coverage from 60 to 80% reduces the time to elimination by 45–55%. The absolute time to elimination is highly uncertain and depends on many factors, such as spatial heterogeneity and importation, therefore here only the relative effects of the different drugs are presented.

### MDA

As for case management, the lowest impact of an MDA is predicted when using a drug with low transmission-blocking effect and with a short duration of protection. Despite this, at low transmission, MDA with a drug with these properties could eliminate infection in low transmission settings (PR_2–10_ of 1%) given a high enough frequency of rounds (here 4 or 6 rounds per year) and high, sustained coverage over a number of years. This result occurs under the simplifying assumption of no importation of infection, and that participation in the MDA at each round is random, rather than that some people are more likely to attend each successive round than others (see [11]). The latter assumption reduces estimated impact of MDA. MDA reduces transmission rapidly compared with case management, with 80% of the total final reduction being achieved within 3, 6 months and 1 year in high, medium and low transmission settings, respectively. However, it must be sustained to maintain these levels of impact [27].

Increasing the duration of protection of the drug is predicted to further reduce clinical cases, with the greatest additional impact predicted at high endemicity. As for case management, the predicted effect increases approximately linearly with increasing duration of protection, though with some diminishing returns when implemented at 6 rounds per year (reflecting a period of almost complete protection) (Fig. 2, bottom row). Notably, administering a drug with a longer duration of protection results in elimination being predicted with fewer rounds per year at low endemicity (PR_2–10_ 1%).

As for case management, increasing transmission-blocking activity from SP-like to ACT-like levels within an MDA was predicted to have a substantial impact on clinical incidence in 0–5 year olds, and there was moderate additional estimated impact when a drug with perfect transmission-blocking activity (ACT + primaquine-like) was used (Fig. 3d–f). At moderate and high transmission, using ACT + primaquine rather than ACT alone was always predicted to have less impact on clinical incidence than increasing the duration of protection of the drug from 3 to 30 or from 30 to 60 days.

Combining increased transmission-blocking activity with a longer duration of protection was predicted to improve MDA impact over a drug which has only one of those properties in the medium and high transmission settings (PR_2–10_ = 10 and 40%, respectively), as was seen in simulations of case management (Fig. 4). In the high transmission setting, the effects of combining drug properties were multiplicative for almost all combinations, as described above for case management. For example a long term intervention of 2 rounds of MDA per year using a drug providing 30 days protection and ACT-like transmission-blocking activity in the high transmission setting achieves an estimated 23% reduction in clinical cases compared with a drug with 3 days protection and SP-like transmission-blocking activity, versus a 17% or a 8% reduction achieved by increased duration of protection alone or transmission-blocking alone, respectively. In this high transmission setting, only at very high intensity of treatment: 6 MDA rounds per year with a 100% transmission-blocking drug and 60 days protection, was the predicted impact (77% reduction) slightly greater than the multiplicative effect of these drug properties alone (71% reduction). In the low-to-medium transmission settings, combined drug properties also acted multiplicatively unless transmission was reduced to very low levels or to zero by MDA. In some scenarios, elimination was possible with combined drug properties, but not by long-acting or by transmission-blocking drugs alone, for example, when 6 rounds of MDA were used per year over the long term in the medium transmission setting (PR_2–10_ = 10%).

## Discussion

This analysis found that the impact of long-lasting or gametocytocidal drug actions varies considerably depending on the local transmission intensity, although the conclusions as to the importance of each drug action in each setting were the same regardless whether anti-malarials were used for case management or for mass treatment. In line with previous analyses [12, 13], this analysis found that as a general trend, clearing blood-stage infections and gametocytocidal action impacts transmission levels most strongly in low endemic settings. Two factors contribute to this: firstly, at high transmission intensity an individual becomes reinfected soon after treatment, so the population-level effect of treatment—preventing the individual from transmitting—is rapidly undone. This explains this trend for both case management and MDA impact. Secondly, with respect to the impact of case management, clinical immunity is common in high transmission areas. Consequently, many inoculations remain asymptomatic and are not treated at all as part of routine case management.

In contrast, long-lasting drugs are predicted to be more useful the higher the transmission intensity, which is backed up by previous analyses and by different models [12, 15]. Prophylaxis was estimated to have almost no effect in low-transmission settings in this model (Fig. 4a) since most people would not be reinfected regardless of whether they are protected or not. When reinoculation is very frequent, however, at high endemicity, post-treatment protection provided by a drug will buy time where the treated individual is uninfected and does not contribute to onward transmission. With increasing duration of protection, the number of re-inoculations averted per person increases proportionally, assuming an approximately constant transmission intensity during the prophylactic period, i.e. no strong seasonal variation or changes in transmission). The relationship of transmission reduction and duration of protection is approximately linear (Fig. 2), except when drugs are taken frequently enough that prophylactic periods overlap. For prioritizing potential long-lasting compounds in terms of transmission reductions, this analysis found that the longer the prophylactic period the better, in a similar way that a vaccine which prevented infection would be preferred to have long lasting action. Additional implications of a long half-life, however, need to be taken into account, such as the risk of promoting resistance spread [28], and the drug tolerability.

The transmission-reducing effect provided by gametocytocidal drugs shows a consistent pattern across the simulated scenarios: an increase from an SP-like drug (no gametocytocidal activity) to an ACT-like drug (moderate to strong transmission-blocking) has a larger impact on transmission (Fig. 3) than an increase from an ACT-like to a perfect transmission blocker (ACT + primaquine-like), although in all settings, both changes make an appreciable impact. This finding shows greater impact of adding primaquine to ACT than some previous analyses [7, 16], which is because the impact of ACT + primaquine on infectiousness had previously been estimated using gametocytaemia measures, whereas it has now been widely shown that this combination has a much larger effect on transmission to mosquitoes [20, 21]. A different model showed more effect of adding primaquine to ACT if combined with a long-acting than short-acting drug, which is similar to our analysis, although our analysis did also find an appreciable effect of ACT + primaquine in the absence of long-lasting drug action [15]. The estimated impact of ACT + primaquine relative to ACT only or SP-like drugs is sensitive to uncertainties: in particular, the duration of untreated malaria infection and contribution to transmission by treated versus untreated infected people [29–31]. For example, if say untreated infections last 200 days and ACT reduces that duration to 5 days (a 97.5% reduction), then the contribution to transmission of treated people is dwarfed by the relatively much longer tail of infectivity in untreated infections. In this scenario, reducing the 5 days to ~0 days of infectiousness, e.g. by adding primaquine to ACT, would achieve little impact. On the other hand, if untreated infections last e.g. for 50 days and ACT treatment reduces that duration to 15 days (a 70% reduction), the contribution of treated individuals to transmission would be more substantial and amenable to further reduction by primaquine. Our analysis assumed a 92% reduction in onward infectivity due to ACT, which allows the addition of primaquine to make a moderate extra impact. The analysis also takes into account differential infectiousness by duration of infection, immunity and by symptomatic versus asymptomatic status, but there is uncertainty in these factors. The duration of infection in endemic areas is very difficult to measure given frequent superinfection and fluctuations in parasite density below diagnostic detection thresholds. It is important to understand these uncertainties given the World Health Organization recommendation in 2012 to add low-dose primaquine to ACT when treating confirmed *P. falciparum* cases in areas close to elimination [32]. Our simulations apply to a situation where ACT are highly effective. Primaquine has been shown to effectively lower onward transmission in an area of artemisinin resistance in the short term, but its duration of impact in infections where asexual parasites recrudesce is less clear [16, 21, 33]. It is important to note that the effect of transmission-blocking in the context of anti-malarial drugs could be significantly lower than the effect of transmission-blocking vaccines. The latter may substantially reduce transmission in all endemicity levels, because the effect of such vaccines are expected to last much longer than the amount of time that the transmission-blocking drug component remains in the blood of the treated patients. Its transmission-blocking action, therefore, affects not only the one infection for which treatment is given (in the case of the drug), but transmission for all future bites within the duration of protection of the vaccine.

There remain some further important uncertainties when modelling treatment impact. Treatment in asymptomatics has not been well studied. This analysis assumed that treatment would cause the same percentage reduction in total onward infectiousness as it does in symptomatic cases, however this percentage could be higher or lower depending on factors such as parasite densities and maturity of gametocytes. The effect of seasonal variation in transmission was not examined in this analysis since this has been covered in detail previously [34] but it is relevant for the impact of treatment. Long-acting drugs for case-management are particularly impactful in areas with highly seasonal transmission because both clinical episodes prompting treatment and infectious bites are concentrated within a short period of time, and therefore the probability of receiving an infectious bite within the prophylactic period is greater. However this effect is not strong in low transmission areas (<5% slide prevalence) because there are too few infectious bites [34]. Seasonal variation in transmission also affects MDA impact: if carried out at the optimal time of year, the lowest transmission season, impact is increased over a non-seasonal setting with the same average annual transmission level [35, 36]. Gametocytocidal drugs increase impact particularly in the low transmission season, in low-to-moderate transmission settings, again because reinfection rates are low at this time of year and individuals who are cleared of infection remain uninfected [14]. A further important consideration is the risk of drug resistance evolution and spread resulting from drugs with different properties. Drug resistance is not currently included in our model, but the role of post-treatment protection and the relative drug pressure exerted by MDA versus case management has been examined in other analyses [16, 28, 37].

This analysis finds that transmission-blocking activity and duration of protection within a single drug regimen combine to increase impact on malaria transmission multiplicatively, i.e. the fact that the drug has transmission-blocking activity does not reduce (or enhance) the relative impact of increasing the duration of protection it provides, and vice versa. This has particularly important implications in medium-to-high transmission settings (for example, where PfPR_2–10_ is between 10 and 40%), where both drug properties are predicted to have important impact on transmission. Combining these two drug properties could, therefore, provide a higher impact of case management or MDA programmes in such settings, enabling them to contribute better towards reducing transmission and potentially elimination.

## Additional file

**Additional file 1.** Additional methods.
