# Supplementary material for: Modelling the benefits of long-acting or transmission-blocking drugs for reducing Plasmodium falciparum transmission by case management or by mass treatment
Source: Malar J. 2017 Aug 16;16:341. doi: 10.1186/s12936-017-1988-4 (PMC5559805; doi:10.1186/s12936-017-1988-4)
Supplement: Supplementary file 1 — Additional file 1. Additional methods. [file 12936_2017_1988_MOESM1_ESM.doc]

Supplementary Information. Modelling the benefits of long-acting or transmission-blocking drugs for reducing Plasmodium falciparum transmission by case management or by mass treatment

Bretscher MT*^1^, Griffin JT^2^, Ghani AC^1^, Okell LC^†1^

^1^ MRC Centre for Outbreak Analysis & Modelling, Department of Infectious Disease Epidemiology, Imperial College, London

^2^ School of Mathematical Sciences, Queen Mary University of London.

## Compartmental Model

For the simulations described in the main manuscript we used a modified, deterministic version of the compartmental model of *P. falciparum* epidemiology as described in [1]. Modifications were described in the main manuscript. Here we give the main modified model equations, but otherwise the model is the same as its previous version (full details available in [1]).

### Human Model

The model is defined by the following partial differential equations, with *t* and *a* representing time and age variables, respectively.

where Λ is the force of infection, and rD, rA, rP and rU denote recovery from the D, A P and U states, respectively. The parameter r­_MDA_ is equal to the proportion of the population treated with MDA, if there is an MDA taking place at time *t*, and otherwise is equal to zero. The remaining parameters are as described in the main text methods and Table 1. The model remains unchanged in other respects. In brief, as well as being stratified by age, each model state is also stratified by exposure, to represent heterogeneity in the rate at which different individuals are bitten. Acquired immunity affects the probability of infection *b* (included in the force of infection Λ), the proportion of infections which develop symptoms *φ* and the probability that an asymptomatically infected individual in state A is detectable by microscopy. The force of infection Λ also varies by age due to increasing biting rates in larger individuals. The mosquito population is explicitly modelled, tracking susceptible, latently infected and infectious mosquitoes. Human infectiousness is modified from the original model to include transmission from the P_S_, P_AU_ and P_DT_ states, as described in the main text.

**References**

1. Griffin JT, Ferguson NM, Ghani AC. Estimates of the changing age-burden of Plasmodium falciparum malaria disease in sub-Saharan Afri**c**a. Nat Commun. 2014; 5:3136.
